# Life history induces markedly divergent insect responses to habitat loss

**DOI:** 10.1111/1365-2656.70117

**Published:** 2025-08-21

**Authors:** Lucas F. Colares, Carlos A. Peres, Cristian S. Dambros

**Affiliations:** ^1^ Programa de Pós‐Graduação em Biodiversidade Animal, Laboratório de Ecologia Teórica e Aplicada, Centro de Ciências Naturais e Exatas Universidade Federal de Santa Maria Santa Maria Rio Grande do Sul Brazil; ^2^ School of Environmental Sciences University of East Anglia Norwich UK; ^3^ Instituto Juruá Manaus Brazil

**Keywords:** Amazon, artificial intelligence, Balbina, body size, deep learning, fragmentation

## Abstract

1. Habitat loss poses a major threat to tropical biodiversity, but its effects on distinct taxa remain unclear. Furthermore, most studies have failed to investigate the effects of habitat loss for taxa with contrasting life histories, potentially underestimating those impacts.
2. Here, using an unprecedented sampling effort, we investigated the effects of forest amount on the diversity, composition and size structure of Amazonian terrestrial and aquatic insects.
3. We sampled the insect fauna across Earth's largest man‐made forest archipelago 36 years after impoundment (Balbina reservoir, Central Amazon, Brazil) using 236 sticky traps placed on forest islands, the open‐water matrix and adjacent continuous forests. Using fivefold cross‐validated computer vision models, we identified and measured 22,471 individual insects. To consider sampling bias on diversity estimation, we used individual‐based rarefaction to partition diversity into components that explained community evenness and regional species pool size. We also applied coverage‐based rarefaction to estimate changes in community composition, reducing potential bias.
4. Low forest amount led to low dominance of terrestrial insects; conversely, it boosted populations of aquatic insects. We report similar effects of forest cover on regional species pool size of aquatic and terrestrial insects, highlighting the importance of large tracts of forest within the landscape to foster diverse communities. Large terrestrial insects were most likely to disperse across the inhospitable floodwater matrix compared to their smaller counterparts.
5. Future studies should consider multi‐taxa approaches to properly quantify impact estimates of land‐use change on biodiversity, which can diverge widely depending on species life history traits. Generalizations and any target conservation action cannot be made without explicitly considering how forest cover can affect species depending on their life history traits.

## Graphical abstract

This study pioneers the use of deep learning to rapidly assess over 22,000 Amazonian insects, revealing life history‐dependent winners and losers from forest loss. It shows that terrestrial insects decline while aquatic insects thrive, with body size influencing dispersal, offering key insights for biodiversity conservation in tropical fragmented landscapes.

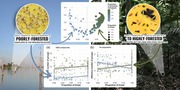

## Resum

1. A perda de habitat representa uma grande ameaça à biodiversidade tropical, mas seus efeitos sobre diferentes táxons ainda permanecem pouco claros. Além disso, a maioria dos estudos não investiga os impactos da perda de habitat em táxons com histórias de vida contrastantes, o que pode levar à subestimação desses efeitos.
2. Neste estudo, utilizando um esforço amostral sem precedentes, investigamos os efeitos da quantidade de floresta sobre a diversidade, composição e estrutura de tamanho de insetos terrestres e aquáticos da Amazônia.
3. Amostramos a fauna de insetos no maior arquipélago florestal artificial do planeta, 36 anos após o represamento (reservatório de Balbina, Amazônia Central, Brasil), utilizando 236 armadilhas adesivas distribuídas em ilhas florestais, na matriz alagada e em florestas contínuas adjacentes. Usando modelos de visão computacional, identificamos e mensuramos 22.471 indivíduos de insetos. Para corrigir vieses amostrais na estimativa de diversidade, utilizamos rarefação para particionar a diversidade em componentes relacionados à equitabilidade das comunidades, ao tamanho regional do conjunto de espécies e para estimar mudanças na composição das comunidades.
4. Baixa cobertura florestal resultou em menor dominância de insetos terrestres, mas, por outro lado, favoreceu populações de insetos aquáticos. Observamos efeitos similares da cobertura florestal sobre o tamanho do conjunto regional de espécies de insetos aquáticos e terrestres, ressaltando a importância de grandes extensões de floresta na paisagem para manter comunidades diversas. Insetos terrestres de maior porte mostraram maior probabilidade de dispersar pela inóspita matriz alagada em comparação com os insetos menores.
5. Estudos futuros devem adotar abordagens multi‐táxon para estimar adequadamente os impactos das mudanças no uso da terra sobre a biodiversidade, já que esses efeitos podem variar amplamente dependendo das características de história de vida das espécies. Generalizações e ações de conservação direcionadas não podem ser formuladas sem considerar explicitamente como a cobertura florestal afeta espécies com diferentes estratégias ecológicas.

## 1 INTRODUCTION

Habitat loss is arguably the most critical threat to insect populations in the Anthropocene (Maxwell et al., 2016; Wagner et al., 2021), leading to extinction rates as high as 1000 times the background rates (He & Hubbell, 2011; Millennium Ecosystem Assessment, 2005). In rapidly declining terrestrial ecosystems in hyper‐diverse tropical regions, this results in changes in species richness (Palmeirim et al., 2019), occupancy (Thornton et al., 2011) and functional composition (Arasa‐Gisbert et al., 2022). However, the true impact of habitat loss on insect population density may have been significantly underestimated, mainly because studies typically fail to consider how different life history traits can affect biodiversity patterns (Stuber & Gruber, 2020). (Jackson & Fahrig, 2015). Studies conducted at small geographic scales (e.g. local scale) tend to find that resource availability and environmental settings required for viable settlement are crucial (Fernández‐Chacón et al., 2014). At broad geographic scales (e.g. landscape scale), isolation and landscape‐scale habitat amount become increasingly important for area‐sensitive taxa but may impact tolerant taxa in different ways (Benchimol & Peres, 2015; Storck‐Tonon & Peres, 2017; van Klink et al., 2020).

habitat loss at the landscape scale results in more isolated remaining habitat patches (Fahrig, 2013; Watling et al., 2020) because of both greater distances from source populations and reduced habitat amount surrounding those patches (Fahrig, 2013; Watling et al., 2020). greater isolation contributes to species‐poor habitat remnants through higher exposure to edge effects (Benchimol & Peres, 2015), genetic drift (MacArthur & Wilson, 1969), disruption in food webs (Palmeirim et al., 2021) and changes in species and trait composition (Arasa‐Gisbert et al., 2022; Benchimol & Peres, 2015). While many taxa are extirpated in this process, some may thrive under those newly modified conditions (McKinney & Lockwood, 1999). Ecomorphological traits largely determine which taxa either ‘win’ or ‘lose’, as they define the nature of interactions with the underlying habitat mosaic (Arasa‐Gisbert et al., 2022; McKinney & Lockwood, 1999; Storck‐Tonon & Peres, 2017). In other words, different taxa could show highly divergent responses to habitat loss. For instance, large‐bodied taxa are often more vagrant, and therefore can easily traverse vast inhospitable matrix areas to reach suitable habitat patches; conversely, smaller, dispersal‐limited and microclimate‐sensitive taxa may be highly susceptible to population declines and local extinction events once confined to isolated habitat remnants (Bie et al., 2012; Storck‐Tonon & Peres, 2017).

Although most organisms often play essential ecological roles, insects surpass other taxa in their importance to ecosystem functioning due to their high diversity, exceptional overall abundance and biomass and distinct life history (Rafael et al., 2012; Wagner et al., 2021; Wong et al., 2019). Insects comprise over 80% of all multicellular species, represent the most diverse phylum of plants and animals and the combined biomass of some 20 quadrillion ants alone exceed that of all wild birds and mammals (Schultheiss et al., 2022). Insects play vital roles in ecosystem functions, such as pollination, pest control and detritus removal, yet concentrate the largest Eltonian shortfall (Rafael et al., 2012; Wong et al., 2019). Although insects have been famously referred to as ‘little things that run the world’ (Wilson, 1987), they are often overlooked in conservation ecology studies due to their unwieldy diversity, relative obscurity and sheer abundance, all of which present intractable sampling and identification challenges. However, time is rapidly running out to understand insect responses to habitat loss and isolation due to long‐term declines in insect populations worldwide, if not ‘Insect Armageddon’ (Wagner et al., 2021).

Here, we investigate community‐wide responses of flying insects characterized by different life history traits to tropical old‐growth forest loss within a vast archipelagic landscape of Central Amazonia. To achieve this, we trained a deep learning network to automatically identify and measure 22,471 individual insects captured on 236 sticky traps distributed across a ~360,350‐ha study area. We classified insects into two major functional groups based on their life cycles: (1) terrestrial insects; and (2) aquatic insects that depend on water during at least one stage of their ontogeny. (Jackson & Fahrig, 2015; Stuber & Gruber, 2020) We expected insect assemblages throughout the wider and often more isolated open‐water matrix of the Balbina archipelago to be, on average, larger‐bodied than their counterparts in highly forested landscapes, regardless of their life history cycles. This assumption is based on previous evidence that large‐bodied flying insects are typically conferred high dispersal capacities (Bie et al., 2012; Greenleaf et al., 2007).

## 2 MATERIALS AND METHODS

### 2.1 Study area

This study was conducted at the Balbina hydroelectric dam archipelago in the Central Brazilian Amazon. This vast archipelago contains 3546 islands ranging in size from 0.194 to ~4878 ha that were created simultaneously following the inundation of 251,216 ha of pristine forest along the Uatumã River in 1986. Forest islands in the reservoir are thus surrounded by a freshwater matrix punctuated by a necromass of old‐growth tree snags rising above the floodwaters, as the vegetation was not cleared prior to the impoundment (Fearnside, 1989). The reservoir reaches 30 m in depth, experiences a mean rainfall regime of 2376 mm/year, a mean annual temperature of ~28°C and the vegetation on the islands is dense, with an average of 143 trees ≥10 cm of diameter at breast height ha^−1^ (Benchimol & Peres, 2015). To mitigate the effects of habitat loss, the adjacent forest areas were protected in 1990 with the creation of the ~940,000 ha Uatumã Biological Reserve, the largest terrestrial protected area of this type in Brazil (ICMBio, 2002).

### 2.2 Insect sampling

**FIGURE 1:**
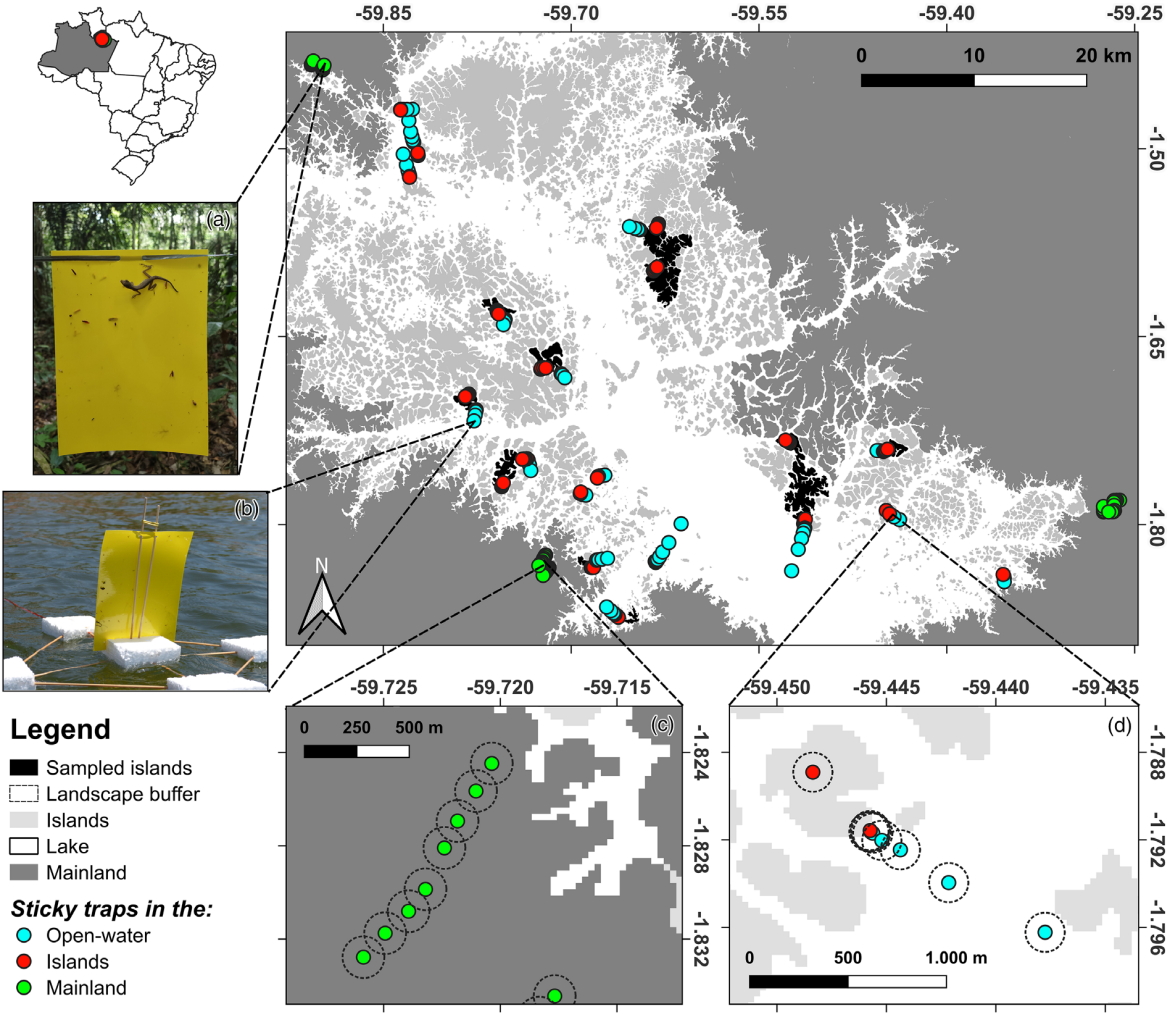
Geographic location of the 236 terrestrial and aquatic sticky traps distributed across 17 widely spaced islands and 3 continuous forest sites within the Balbina archipelagic landscape, Brazil. Main panel illustrates the positioning of sticky traps at (a) island (red circles) and continuous forest sites (green circles) and (b) the open‐water matrix of the reservoir lake (blue circles). Bottom panels illustrate the spacing between sticky traps along (c) the forest and (d) open‐water transects as well as the 100‐m landscape size used to extract forest cover.

From October to November 2021, we distributed 236 rectangular (20 cm × 15 cm) double‐sided yellow sticky traps (E‐Know Fruit Fly Outdoor© traps; Figure 1a) on 17 forest islands (72 traps), the wider open‐water matrix (119 traps) and 3 continuous upland (*terra firme*) forest sites (45 traps) in the mainland surrounding the Balbina reservoir (Figure 1). Each trap was exposed over a period of 24 consecutive hours before it was collected to avoid overcrowding. Then, traps were photographed on both sides and preserved in 96% ethanol. Each trap therefore had a two‐sided trapping area of 600 cm^2^, which amounted to a total effort of 14,400 cm^2^ sampled per hour over each day/night. The 24‐h exposure period was more than sufficient to saturate the surface area of several sticky traps (Appendix S1). On each island, we installed, wherever possible, five sticky traps every 100 m along an edge‐to‐core forest transect from the island perimeter to 500 m inland. For islands larger than 500 ha, we sampled two different 500 m transects on opposite sides of the island. For islands smaller than 125 ha, we sampled a single edge‐to‐core transect containing five evenly spaced traps. Finally, for islands smaller than 2.5 ha, we placed three evenly spaced traps along an edge‐to‐edge transect. For the open‐water matrix, we designed and built a floating sticky trap model, consisting of five polystyrene sheets perforated by wooden sticks (see Figure 1b) to sample a linear aquatic transect comprised of eight floating traps that were spaced apart from each focal island along a quasi‐log distance scale (i.e. 10, 30, 80, 200, 500, 1000, 2000 and 4000 m; Figure 1). These traps were tightly anchored to the bottom of the reservoir lake, thereby remaining stationary. At each mainland continuous forest site, we distributed five evenly spaced traps along eight 500 m forest transects, with each forest transect 500 m apart from each other. Our study required no ethics approval, following the guidelines of Brazilian law number 11,794/2008. All permits for biological sampling in conservation units were appropriately granted by the Chico Mendes Brazilian Institute for Biodiversity Conservation under licence #79466.

### 2.3 Image post‐processing and taxonomic identification

We used a deep learning model network to perform an object detection task on standardized images derived from all sticky traps. Using a DMC‐FZ60 Panasonic camera, we photographed both sides of the 236 sticky traps at a fixed distance from each trap panel, resulting in 472 photos. We implemented an automatic image segmentation pipeline, as described in Appendix S2, to differentiate between the yellow background (i.e. the sticky‐trap) and the foreground (i.e. potential individual insects) of each image. We further segmented these 472 images into 14,090 smaller pieces to zoom into all individual insects (see examples at Appendix S3) captured and avoid the problem of small object detection commonly encountered in object detection tasks (Tong et al., 2020). From these 14,090 sub‐images, we selected 999 to train and validate our object detection model. For those 999 images, we drew a boundary box around each individual insect, extracted its x, y coordinate and assigned its taxonomic order or subfamily in a process termed ‘annotation’.

In total, we annotated 1568 individuals and assigned their higher‐level taxonomic resolution as follows: Blattaria (i.e. cockroaches; 3 ind.), Brachycera (i.e. midge flies; 213 ind.), Coleoptera (i.e. beetles; 205 ind.), Ephemeroptera (i.e. mayflies; 213 ind.), Hemiptera (i.e. mostly cicadas; 269 ind.), Hymenoptera (bees and wasps; 217 ind.), Isoptera (i.e. termites; 16 ind.), Lepidoptera (i.e. butterflies; 29 ind.), Nematocera (i.e. mosquitoes; 298 ind.), Odonata (i.e. dragonflies; 22 ind.), Orthoptera (i.e. grasshoppers; 7 ind.), Plecoptera (i.e. stoneflies; 12 ind.) and Trichoptera (i.e. caddisflies; 34 ind.). We used high‐level taxonomic resolution to assess biodiversity to ensure a rapid and spatially comprehensive flying insect inventory. This is not necessarily a limitation; as previous studies have shown congruent results with lower taxonomic resolution such as genera and species (de Oliveira et al., 2020).

To train and validate our deep learning model, we divided 999 annotated images into training and test datasets, with 798 images (80%) allocated for training and 201 images (20%) for testing. Importantly, both datasets contained representative images from all taxonomic groups, ensuring balanced validation. We trained and performed fivefold cross‐validation using the extra‐large variant of the YOLO object detection model (YOLOv8; Redmon et al., 2016) for 250 epochs, with a batch size of 50 and an input image size of 640 × 640 pixels. Epoch size was guided by the ‘patience’ metric embedded in the Ultralytics Python package (Jocher et al., 2023). The use of fivefold cross‐validation ensured that each of the 999 images contributed to both training and testing, maximizing classification accuracy. Using these trained YOLOv8 models, we then conducted inference on all 472 photos obtained from sticky traps. Because small insects can be difficult to detect on large yellow sticky cards, we applied a Sliced Aided Hyper Inference (SAHI; Akyon et al., 2022) approach to improve detection. SAHI enabled inference on smaller 500 × 500‐pixel tiles of the original high‐resolution images (4608 × 3465 pixels).

After performing inference, we calculated the Intersection over Union (IoU) for all detected objects across the five model folds. When multiple inferences overlapped with an IoU higher than 0.5, only the inference with the highest confidence score was retained. We then conducted a sensitivity analysis across all five models by simulating a range of confidence thresholds (0 to 0.99). At each threshold, we calculated performance metrics including recall (i.e. proportion of insects correctly identified), precision (i.e. proportion of true‐positives) and the *F*1 score (i.e. harmonic mean of accuracy and recall). For each taxonomic group and cross‐validation fold, we selected the optimal confidence threshold based on the highest accuracy value. This approach minimized false‐positives while maximizing the retention of correct inferences (Appendices S2 and S3). We excluded predictions for non‐insect groups (e.g. spiders), as well as groups that either achieved their highest precision at a confidence threshold of 0 or had fewer than 30 training images, to reduce misclassification rates. This led to the removal of Odonata, Plecoptera, Blattaria, Isoptera, Lepidoptera, Araneae and Orthoptera. All models were trained using an © NVIDIA A100 GPU.

Following Rafael et al. (2012), the remaining seven taxonomic groups identified by our YOLOv8 deep learning model were classified as either terrestrial or aquatic based on the ontogeny of the majority species within each biological group. This classification allowed us to identify the role of life history on the effects of forest cover. Accordingly, if an insect experienced at least one aquatic life stage during its ontogeny, then it was assigned to an aquatic lifecycle; otherwise, it was assigned to a terrestrial lifecycle. We classified the following insect taxa as aquatic: Ephemeroptera, Nematocera and Trichoptera. We classified the following taxa as terrestrial: Brachycera, Coleoptera, Hemiptera and Hymenoptera.

### 2.4 Biological metrics

As variation in species diversity can result from changes in either total abundance (the N‐component) or the regional species pool size (the SAD‐component; Engel et al., 2022), we partitioned diversity into these two components. This approach uses the effective number of species transformation derived from individual‐based rarefaction curves, which are independent of sample size (Engel et al., 2022). To construct our individual‐based rarefaction curves, we used a subsample size equal to the minimum number of individuals found in any sticky trap across the entire dataset (i.e. four individuals). Additionally, we included only those traps in which the total number of individuals exceeded the number of biological groups represented. We present results on the effects of forest cover for both diversity components (i.e. N and SAD) across the life history groups (i.e. terrestrial and aquatic insects). For further mathematical details on the calculation of the N and SAD components, see Engel et al. (2022).

To estimate changes in community composition and abundance, we performed two principal coordinates analyses (PCoA) to summarize variation in the occurrence and abundance of all biological groups (Legendre & Legendre, 2012). For abundance data, we used the Bray–Curtis distance, while for occurrence data we used the Jaccards distance (Legendre & Legendre, 2012). It is worth noting that PCoA has certain limitations when used to represent community composition, as it may not fully capture the underlying data structure. To address this, we complemented our analysis with a coverage‐based rarefaction approach to estimate beta diversity using the β_C_ index (Engel et al., 2021). This method applies null models to remove biases related to sample completeness and regional species pool size. β_C_ was calculated separately for terrestrial and aquatic life cycle groups, allowing us to assess which group contributed most to compositional turnover across the Balbina archipelago. To ensure comparability between life cycle groups in β_C_ calculations, we first computed the recommended maximum coverage (MC) value for each life cycle group and then standardized both MC values to the minimum value minus 0.01 (Engel et al., 2021). While calculating β_C_, we used 99 samples per subset (from a total of 236 traps) and performed 9999 randomizations of the null model.

To represent the abundance of each taxon, we summed the occurrences of all individuals on both sides of each sticky trap, which resulted in a total abundance value for each trap for each of the 13 insect taxonomic groups. We measured the area of the bounding box (in pixels) for each inference and calculated the maximum bounding box area at each trap as a proxy of the largest individual recorded per taxon per trap (i.e. hereafter, body size). We tested for phylogenetic signal in the residuals of a global body size GAM model, structured as follows: *size* ~ *forest* * *taxa*, using the lambda method (i.e. Blomberg et al., 2003) and found no support for phylogenetic signal (lambda = 0.000041, logL_lambda_ = −41.26, LR_lambda = 0_ = −0.0001, *p* = 1; following Misof et al., 2014 as reference to build a phylogenetic tree for insect groups).

### 2.5 Landscape metrics

To estimate how habitat loss could affect the abundance and size structure of insect assemblages, we used classified georeferenced Landsat ETM+ scenes with a 30 m resolution from the Balbina archipelago for the year 2021, sourced from the MapBiomas database (Souza et al., 2020). We calculated the proportion of forested pixels at 100 m sized circular buffers centred on each sticky trap (Figure 1c,d). We chose the 100‐m sized buffer as previous studies pointed to an acceptable buffer size for invertebrates, considering their limited dispersal in comparison to many vertebrate taxa (Jackson & Fahrig, 2015). However, we acknowledge that other buffer sizes can be relevant depending on each insect group's intrinsic dispersal abilities (Jackson & Fahrig, 2015; Miguet et al., 2016). We calculated forest cover for the group of pixels corresponding to native forested vegetation in the landscape, including forest formation, savannah formation, mangrove, floodable forest and wooded sandbank vegetation (Souza et al., 2020). The MapBiomas database consists of a comprehensive collection of land use classification in 29 different classes across Brazil and can be retrieved as GeoTIFF files from their online platform at https://brasil.mapbiomas.org/ (Souza et al., 2020).

### 2.6 Data analysis

We assessed the effects of forest cover on insects using generalized additive models (GAMs) at two levels: lifecycle and taxonomic. At the lifecycle level, we examined how forest cover influences the N‐ and SAD‐components of diversity and the composition of aquatic and terrestrial insects, represented by the first axis of abundance‐ and occurrence‐based PCoAs. Models included the interaction between lifecycle category and forest cover as fixed effects. At the taxon level, we evaluated the effects of forest cover on the abundance and body size of seven insect groups (caddisflies, mayflies, mosquitoes, flies, beetles, cicadas and bees/wasps) across all traps. Due to overlapping trap‐scale buffers and potential spatial autocorrelation (Figure 1c,d), we included smoothed trap coordinates in the GAMs (Legendre & Legendre, 2012) and verified spatial independence using Moran's *I* (Moran, 1950; Appendix S4). For model complexity, we tested 20 values for the number of knots (*k* = 1–20) and selected the model with the lowest AIC.

In total, we ran 80 GAMs at the lifecycle level (20 *k*‐values × 4 response variables: N, SAD, abundance‐ and occurrence‐based composition) using the structure: y ~ forest cover * lifecycle + s(*coordinates*). At the taxon level, we ran 280 GAMs (20 *k*‐values × 7 taxa × 2 response variables: abundance and body size) using: *y* ~ *forest cover* + *s*(*coordinates*). We used Gaussian distribution for GAMs involving diversity components and PCoAs; Poisson for taxon‐level abundance; and quasi‐Poisson for body size.

All data extraction, calculations and statistical analyses were performed in R (R Development Core Team, 2020), with graphical outputs generated using the *ggplot2* package (Wickham, 2011). We trained our fivefold YOLOv8 deep learning models in Python (Van Rossum & Drake Jr, 1995) using *Ultralytics* (Jocher et al., 2023) and *sahi* (Akyon et al., 2022) libraries. All R and Python code, along with the associated biological and landscape data, are available in a public GitHub repository (Colares, 2025c). Full‐resolution images from the 472 sticky traps (Colares, 2025d), the fivefold image dataset used for training (Colares, 2025a) and the trained models for insect identification (Colares, 2025b) are available on FigShare.

## 3 RESULTS

We identified and measured a total of 22,471 individual insects, of which 68.51% were mosquitoes (confidence threshold (*mCT*) = 0.88 mean ± 0.05 SD; mean average precision at 0.5 IoU (*mAP*_*0.5*_) = 0.79 mean ± 0.03 SD; precision (*Pr*) = 1 ± 0; recall (*R*) = 0.37 ± 0.17; *F*1 = 0.52 ± 0.21), 11.85% were mayflies (*mCT* = 0.92 ± 0.03; *mAP*_0.5_ = 0.72 ± 0.08; *Pr* = 0.98 ± 0.05; *R* = 0.23 ± 0.17; *F*1 = 0.34 ± 0.23), 7.18% were cicadas (*mCT* = 0.94 ± 0.04; *mAP*_0.5_ = 0.81 ± 0.03; *Pr* = 0.98 ± 0.04; *R* = 0.25 ± 0.27; *F*1 = 0.34 ± 0.27), 5.15% were flies (*mCT* = 0.93 ± 0.02; *mAP*_0.5_ = 0.51 ± 0.26; *Pr* = 0.93 ± 0.11; *R* = 0.26 ± 0.13; *F*1 = 0.38 ± 0.14) and 3.82% belonged to beetles (*mCT* = 0.93 ± 0.02; *mAP*_0.5_ = 0.82 ± 0.09; *Pr* = 1 ± 0; *R* = 0.47 ± 0.26; *F*1 = 0.61 ± 0.23). Hymenopterans (*mCT* = 0.94 ± 0.02; *mAP*_0.5_ = 0.67 ± 0.08; *Pr* = 0.88 ± 0.19; *R* = 0.22 ± 0.24; *F*1 = 0.28 ± 0.26) and caddisflies (*mCT* = 0.87 ± 0.05; *mAP*_0.5_ = 0.69 ± 0.07; *Pr* = 0.95 ± 0.11; *R* = 0.4 ± 0.15; *F*1 = 0.54 ± 0.13) represented together 3.47% of all insect records in our study (for the remaining validation metrics of our YOLOv8 models, see Appendix S2).

### 3.1 Lifecycle‐level responses

**FIGURE 2:**
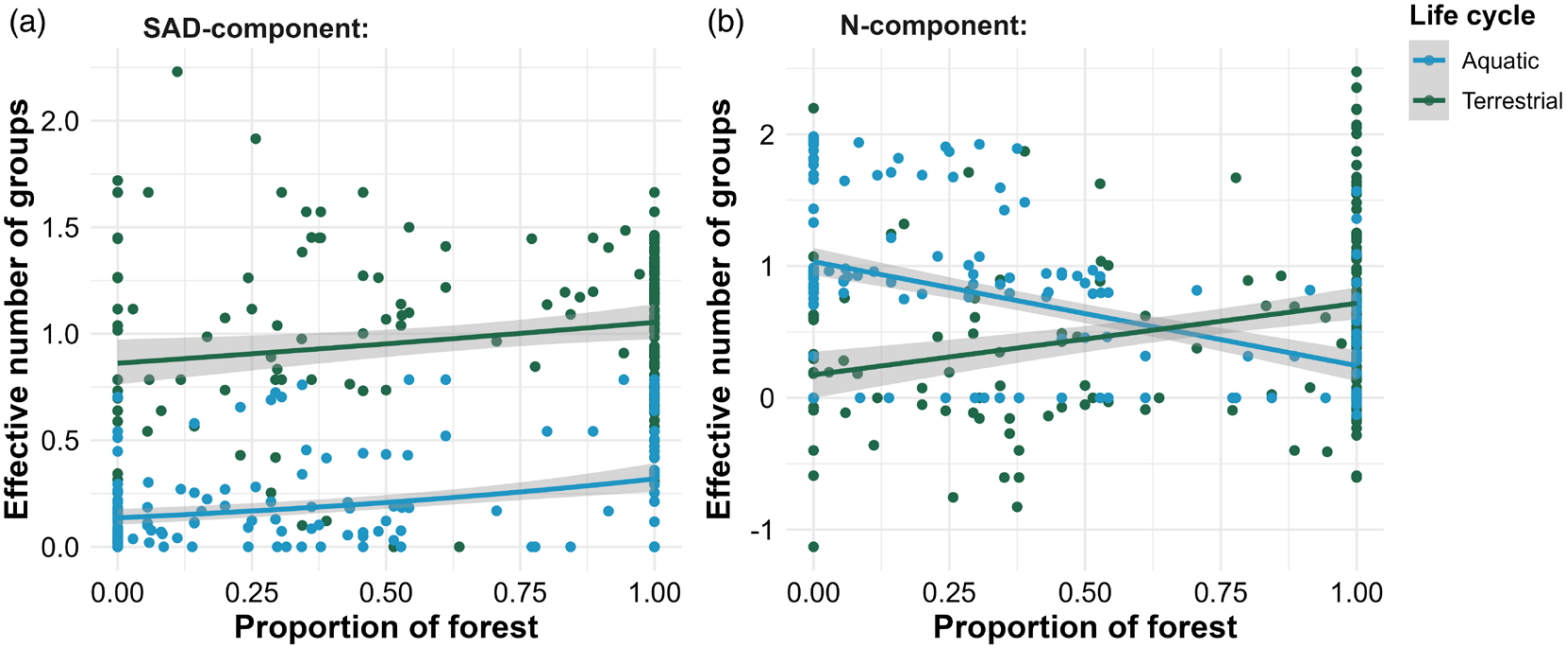
Effects of landscape‐scale proportion of forest cover on the effective number of groups (as returned by the individual‐based rarefaction) for the (a) SAD and (b) N components of diversity of both lifecycle groups (top panels). Green and blue lines and circles represent terrestrial and aquatic insects, respectively. Lines represent the direction of associations and grey shading their 95% confidence intervals.

Forest amount increased the SAD component of insects regardless of their life cycle (*b* = 0.425, standard error = 0.093, *t* = 2.74, *p* < 0.01; Figure 2a). Conversely, forest amount had a positive effect on the N‐component of terrestrial insects, but the opposite effect on the N‐component of aquatic taxa (*b*_terrestrial_ = 1.36, standard error_terrestrial_ = 0.14, *t*_terrestrial_ = 9.72, *p*_terrestrial_ < 0.01; *b*_aquatic_ = −0.93, standard error_terrestrial_ = 0.1, *t*_aquatic_ = −8.97, *p*_aquatic_ < 0.01; Figure 2b). Forest cover and lifecycle explained 50.9% and 28% of the deviance in the SAD and N components of insect diversity, respectively (Figure 2c,d; Appendix S5).

**FIGURE 3:**
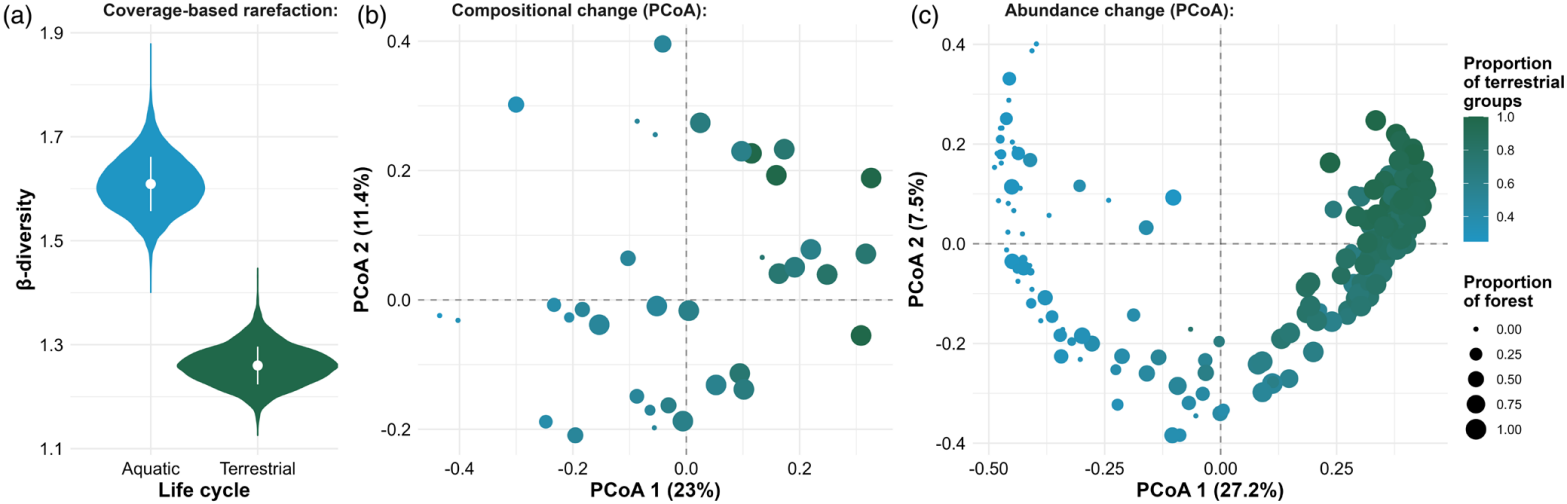
Effects of landscape‐scale forest cover on insect community composition. Panel (a) show the distribution of the 9999 β_C_ values for both terrestrial (green violins) and aquatic (blue violins) groups returned after coverage‐based rarefaction. In panel (a), the white point represents the mean estimation, while the lines represent its standard deviation. Panels (b and c) shows how (b) occurrence‐based and (c) abundance‐based composition changes from a terrestrial‐dominated to an aquatic‐dominated community (from left to right along the *x* axis) depending on the proportion of forest cover in a 100‐m landscape. In panels (b and c), circle size varies depending on forest cover around each trap, and circle colour represents the proportion of terrestrial (b) groups and (c) individuals captured in that specific trap.

We evidenced marked changes in abundance‐based and occurrence‐based community composition driven by forest cover (adjusted *R* ^2^_abundance‐based_ = 0.77, *b*_abundance‐based_ = 0.74, standard error_abundance‐based_ = 0.03, *t*_abundance‐based_ = 21.91, *p*_abundance‐based_ < 0.01; adjusted *R* ^2^_occurrence‐based_ = 0.43, *b*_occurrence‐based_ = 0.29, standard error_occurrence‐based_ = 0.02, *t*_occurrence‐based_ = 11.85, *p*_occurrence‐based_ < 0.01; Figure 3b,c; Appendix S5). When forest cover increases in the landscape, insect occurrence‐based (Figure 3b) and abundance‐based (Figure 2c) composition shifts from a community dominated by aquatic insects to a terrestrial insect‐dominated community. Coverage‐based β‐diversity evidenced that such turnover is mainly due to changes in the distribution of aquatic insects rather than the terrestrial groups (Figure 2a).

### 3.2 Taxon‐level patterns

**FIGURE 4:**
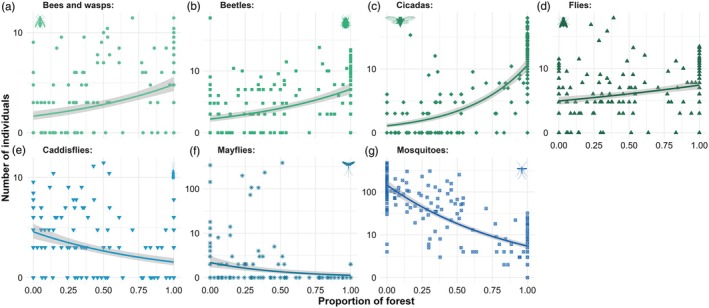
Taxon‐specific responses to proportion of landscape‐scale forest cover are represented for numerical abundance (in log_10_ *x*). Line colours and symbol shapes represent different taxa: closed circles are (a) bees and wasps, squares are (b) beetles, diamonds are (c) cicadas, triangles are (d) flies, upside‐down triangles are (e) caddisflies, asterisks are (f) mayflies, and open squares are (g) mosquitoes. Green and blue lines and symbols represent (a–d) terrestrial and (e–g) aquatic insects, respectively, and grey shading their 95% confident intervals.

The abundance of ubiquitous strictly terrestrial taxa (i.e. flies, beetles, cicadas, bees/wasps) was positively affected by overall forest cover (Appendix S6; Figure 4a–d). Conversely, aquatic insect abundance was negatively affected by higher forest cover (Figure 4e–g; Appendix S6). Forest cover explained 73%, 69%, 25%, 14%, 9%, 2% and 1% of the deviance in the abundance of cicadas, mosquitoes, caddisflies, bees/wasps, beetles, mayflies and flies, respectively (Figure 4).

**FIGURE 5:**
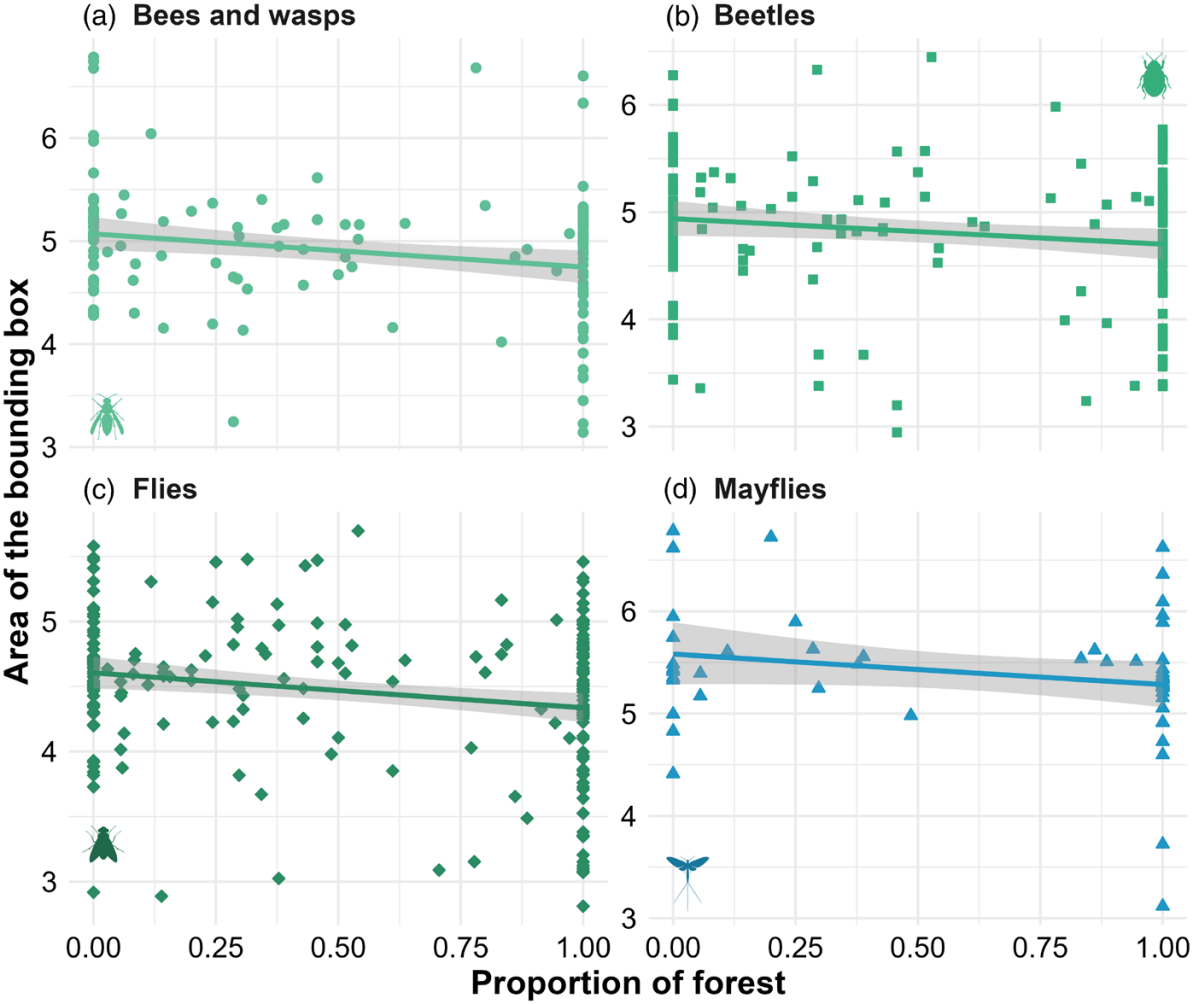
Taxon‐specific responses to the proportion of forest cover at the landscape scale are illustrated in terms of body size, measured as the area of the inference bounding box returned by the YOLOv8 models. Line colours and symbol shapes denote different taxa: green circles are (a) bees and wasps; green squares are (b) beetles; diamonds are (c) flies; and triangles are (d) mayflies. Green and blue lines and symbols represent terrestrial and aquatic insects, respectively, with shaded grey areas indicating their 95% confidence intervals.

We evidenced taxon‐specific body size responses to forest cover (Appendix S6; Figure 5c). Bees/wasps (Figure 5a; *R* ^2^_adj_ = 0.08), beetles (Figure 5b; *R* ^2^_adj_ = 0.01), flies (Figure 5c; *R* ^2^_adj_ = 0.02) and mayflies (Figure 5d; *R* ^2^_adj_ = 0.09) responded negatively to forest cover, and were all larger in highly deforested landscapes. We found no significant effects of forest cover on the body size of caddisflies, cicadas and mosquitoes (Appendix S6).

## 4 DISCUSSION

### 4.1 Lifecyle defines responses to forest loss

Here, we shed light on the importance of considering multiple taxa and their individual life history traits in identifying ‘winners’ and ‘losers’ due to forest loss in terms of population size, taxonomic diversity and composition responses. Although some taxa may thrive in the aftermath of habitat change, while other taxa may decline (McKinney & Lockwood, 1999), most terrestrial studies have focused on taxa for which forest loss has a detrimental impact on biodiversity (Arasa‐Gisbert et al., 2022; Benchimol & Peres, 2015; Storck‐Tonon & Peres, 2017). In the aftermath of tropical forest loss induced by a major dam, the Balbina archipelago experienced a marked surge in aquatic insect populations, while the terrestrial insect population within the vast freshwater matrix succumbed to a sharp decline, which is now evident after 36 years of impoundment.

The effects of forest loss on insect populations in the Balbina archipelago appear to reflect a ‘more‐individual’ effect (Engel et al., 2021, 2022), which suggests that observed diversity patterns may result from passive sampling during biological surveys. Under this effect, communities with higher local abundance tend to capture a larger portion of the regional species pool simply due to the greater number of individuals sampled (Engel et al., 2022). The contrasting responses of aquatic and terrestrial insects with respect to the *N* component of diversity provide evidence for this phenomenon. In landscapes with low forest cover, insect communities are numerically dominated by aquatic taxa. We suggest that such a pattern arises from the dominance of a few species that have their abundance fostered by the alterations promoted by river impoundment (see Walker, 2009). Conversely, highly forested landscapes are dominated by terrestrial insects, probably due to increased availability of food resources, nesting substrates and microhabitats that support larger terrestrial populations (Shipley et al., 2023; Storck‐Tonon & Peres, 2017; Wagner et al., 2021).

Despite these divergent abundance patterns, both aquatic and terrestrial groups exhibited similar responses regarding the SAD component of diversity, which is influenced by the size of the regional species pool (Engel et al., 2022). Thus, forest loss appears to increase overall diversity for both life cycle groups, aligning with findings from previous studies (Palmeirim et al., 2021; Storck‐Tonon & Peres, 2017; Watling et al., 2020). While the shift from terrestrial to aquatic dominated communities may be expected in landscapes where habitats have been converted from terrestrial to freshwater, a global meta‐analysis showed a general long‐term rise in aquatic insect populations and a gradual decline of terrestrial insects in unprotected temperate regions, regardless of habitat type (van Klink et al., 2020). While the results from previous studies in tropical regions remain inconclusive, our study provides compelling evidence that this pattern likely holds true for tropical areas experiencing forest loss as well. Despite this shift from terrestrial to aquatic insects, the few taxa that benefit from forest loss often fail to exert the same ecological roles of their many predecessor ‘loser’ counterparts, leading to shifts in functional composition and, consequently, ecosystem dynamics (Liu et al., 2018).

This pattern of functional compositional shift is likely occurring in the Balbina Reservoir, where our findings revealed clear differences in taxonomic composition along a gradient of forest cover in the surrounding landscape. Forest loss following river impoundment has triggered a marked turnover in insect communities, with aquatic insect groups increasingly replacing terrestrial counterparts in areas with low forest cover. In highly forested landscapes, terrestrial invertebrates play essential ecological roles, providing services such as pollination, pest control, dung removal and regulation of other populations (Greenleaf et al., 2007; Storck‐Tonon et al., 2020; Storck‐Tonon & Peres, 2017). However, the extensive freshwater matrix created by the reservoir acts as a dispersal barrier for many terrestrial insect species, limiting their movement and reducing the spillover of these critical ecosystem functions into deforested areas (Liu et al., 2018; Matthews, 2021; Shipley et al., 2024). Otherwise, the dominance of a few opportunistic aquatic taxa in poorly forested landscapes, particularly mosquitoes, could lead to the proliferation of tropical diseases such as malaria and dengue fever (Millennium Ecosystem Assessment, 2005; Yi et al., 2014). This community turnover is primarily driven by changes in the abundance of aquatic insects, which can be seen as ecological ‘winners’ in the context of forest loss in the Balbina landscape. Unlike most terrestrial groups that lose habitat as deforestation occurs, these aquatic taxa gain new breeding grounds and expand their populations in the altered environment.

We showed that forest loss exerts divergent impacts on flying insects depending on a key morphological trait associated with many ecosystem functions: body size. While large‐bodied vagrant mayflies, flies, beetles, wasps, and bees were able to traverse the vast open‐water matrix over linear distances of as much as 4 km, their smaller‐bodied and presumably less vagile counterparts were restricted to the vicinities of dense forest cover (Figure 5). This indicates an ‘upsizing’ effect promoted by the hostile open‐water matrix to at least the forest affiliated insect fauna and a few groups of aquatic insects (i.e. mayflies). As such, large‐bodied insects accrued a landscape‐scale advantage in overcoming habitat isolation, presumably because flight endurance is positively associated with body size, wingspan and thorax muscle mass (Bie et al., 2012; Greenleaf et al., 2007; Storck‐Tonon & Peres, 2017).

Conversely, smaller‐bodied terrestrial insects are largely stranded on forest islands and mainland sites, reflecting a pattern previously shown for Euglossine bees (Storck‐Tonon & Peres, 2017). The loss of small insects may exert further effects on ecosystem functioning, as many small beetles, bees and wasps play important roles in nutrient cycling, pollination and population control through parasitism, respectively (Rafael et al., 2012; Shipley et al., 2024; Wong et al., 2019). This highlights the importance of large tracts of forest within the landscape in maintaining the ecosystem services provided by insects across their entire size spectrum (Storck‐Tonon & Peres, 2017).

## 5 CONCLUSIONS

We unveil the nuanced impact of forest loss at a tropical freshwater–land interface on hyper‐diverse tropical insect communities, indicating life history‐dependent outcomes. We employed a methodological design that enabled rapid and spatially extensive sampling of insect communities using a low‐cost sampling method. Using deep learning, we achieved high‐resolution taxonomic identification and body size measurements for more than 22,000 insect individuals in record time (i.e. less than 6 months from field collection). While intrinsic limitations of our methods are evident, we provide compelling results on insect community assembly in response to forest loss.

Our results evidence that while the reduction of forest amount due to human disturbance decreased the number of terrestrial organisms, the number of aquatic insects increased. We also show that regional species pool size increases for both life cycle groups in highly forested landscapes, underscoring the importance of native habitat on the promotion of diverse insect communities. Larger‐bodied insects exhibit higher dispersal capacity. As a corollary, the incidence of small‐bodied insects with important ecological roles is potentially reduced in small, isolated islands or forest patches. Finally, we demonstrate that habitat loss has a strong effect on insect populations locally, at small landscape scales (i.e. 100 m). Future studies should build on our findings and recent advances in computer vision to move beyond current methods, applying rapid, low‐cost insect surveys at finer taxonomic resolutions (given the tremendous species diversity with the seven main groups we approached here). These findings advance our current understanding of the intricate interplay between habitat loss, species traits and ecosystem dynamics in tropical forest landscapes and pave the way for effective insect conservation efforts in our ever‐changing world.

## AUTHOR CONTRIBUTIONS

All authors conceived the ideas, designed methodology, contributed critically to the drafts and gave final approval for publication; LFC collected and analysed the data and led the writing of the manuscript. Our study brings together solely Brazilian authors, two of whom were born in the Amazon. All authors were involved early in the research and study design to ensure that the diverse perspectives they represent were considered from the outset. When relevant, literature published by scientists from the region was cited. The local community and stakeholders of Balbina village participated in the field campaigns, and the first author carried out outreach initiatives at the local high school during these campaigns.

## CONFLICT OF INTEREST STATEMENT

The authors declare no conflicts of interest.

## Supporting information


**Appendix S1.** Sticky trap field images.
**Appendix S2.** Description of image treatment and object detection model.
**Appendix S3.** Samples of insect taxonomic identifications by our custom deep learning model.
**Appendix S4.** Moran's *I* result.
**Appendix S5.** Generalized Additive Models coefficients for the two diversity components (i.e., SAD and N) and composition of different lifecycle groups at nine landscape sizes.
**Appendix S6.** Generalized Additive Mixed Models coefficients for abundance and body size of different taxonomic groups at nine landscape sizes.

## Data Availability

Processed data available from the Zenodo repository (https://doi.org/10.5281/zenodo.15238078; Colares, 2025c). We also provide the raw images of the sticky traps (https://doi.org/10.6084/m9.figshare.23823591; Colares, 2025d), the fivefold dataset with insect images used for deep learning training (https://doi.org/10.6084/m9.figshare.28688198; Colares, 2025a) and the five trained models to conduct inference on insect taxonomy using deep learning (https://doi.org/10.6084/m9.figshare.28820993; Colares, 2025b).
