# Supplementary material for: Life history induces markedly divergent insect responses to habitat loss
Source: J Anim Ecol. 2025 Aug 21;95(1):54–64. doi: 10.1111/1365-2656.70117 (PMC12775549; doi:10.1111/1365-2656.70117)
Supplement: Supplementary file 1 — Appendix S1. Sticky trap field images. Appendix S2. Description of image treatment and object detection model. Appendix S3. Samples of insect taxonomic identifications by our custom deep learning model. Appendix S4. Moran's I result. Appendix S5. Generalized Additive Models coefficients for the two diversity components (i.e., SAD and N) and composition of different lifecycle groups at nine landscape sizes. Appendix S6. Generalized Additive Mixed Models coefficients for abundance and body size of different taxonomic groups at nine landscape sizes. [file JANE-95-54-s001.docx]

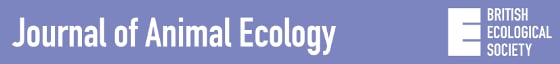


**Journal:** Journal of Animal Ecology

**Supporting information for:** Life history induces markedly divergent insect responses to habitat loss

**Authors:** Lucas F. Colares^1^, Carlos A. Peres^2,3^, Cristian S. Dambros^1^

**Affiliations:** ^1^Programa de Pós-Graduação em Biodiversidade Animal, Laboratório de Ecologia Teórica e Aplicada, Centro de Ciências Naturais e Exatas, Universidade Federal de Santa Maria, Santa Maria – RS, Brazil. ^2^School of Environmental Sciences, University of East Anglia, Norwich, UK. ^3^Instituto Juruá, Manaus, Brazil

**This file includes:**

Appendix S1 – Sticky trap field images (page 2).

Appendix S2 – Description of image treatment and object detection model (page 3).

Appendix S3 – Samples of insect taxonomic identifications by our custom deep learning model (page 10).

Appendix S4 – Moran’s I result (page 11).

Appendix S5 – Generalized Additive Models coefficients for the two diversity components (i.e., SAD and N) and composition of different lifecycle groups at nine landscape sizes (page 15).

Appendix S6 – Generalized Additive Mixed Models coefficients for abundance and body size of different taxonomic groups at nine landscape sizes (page 21).

## Appendix S1. Sticky traps after 24h of exposure in the Balbina reservoir, Central Amazon.

##
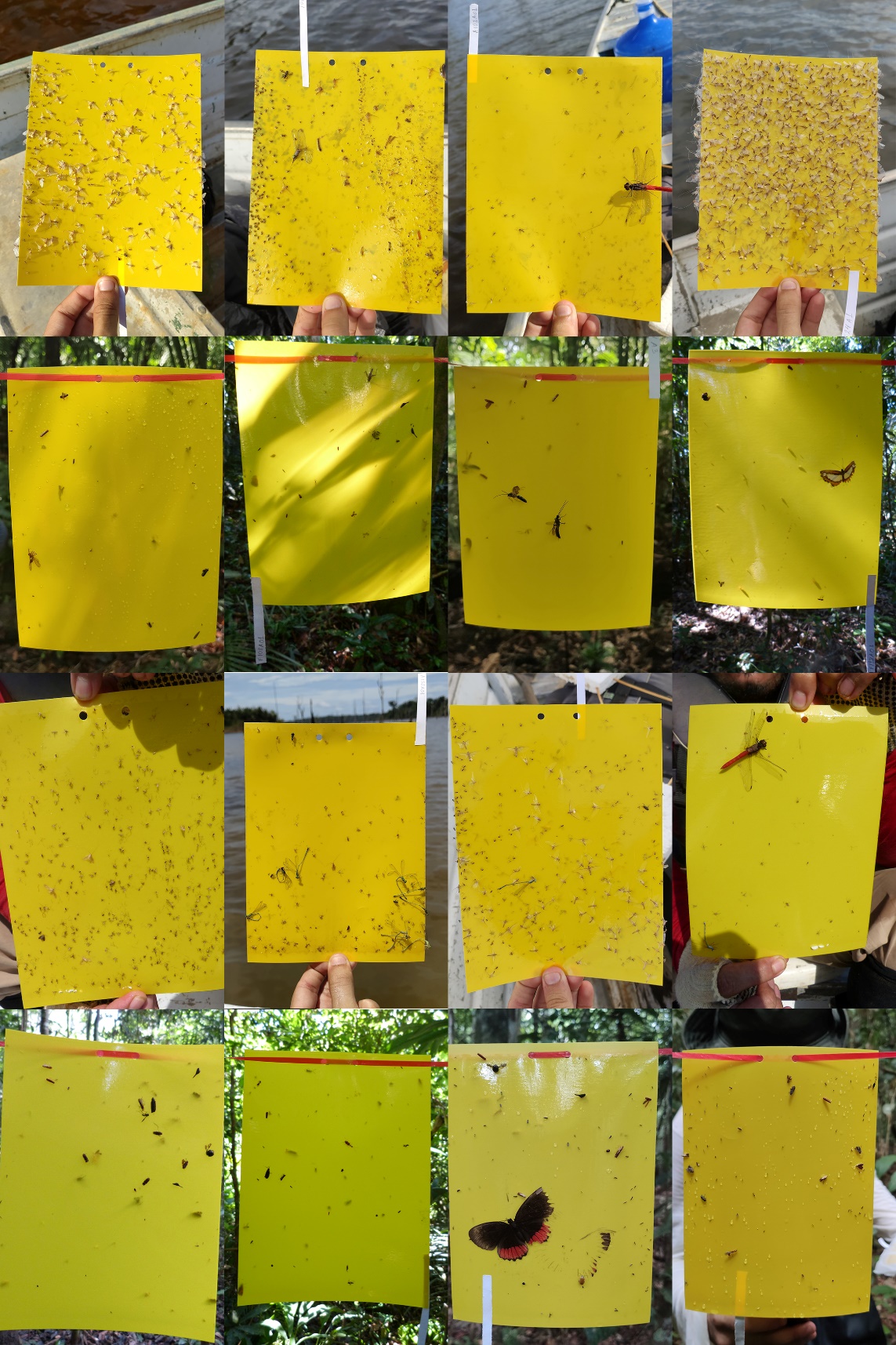


**Figure S1.** Examples of sixteen yellow sticky traps after 24h of exposure in the Balbina archipelago, Central Amazon.

## Appendix S2. Image treatment and object detection model descriptions.

Initially, we converted the 472 colour images to grayscale and blurred them to soften the edges of the foreground. Then, we applied an Adaptive Threshold to binarize the images. The Adaptive Threshold method calculates the background threshold of an image for smaller regions, resulting in different background thresholds for each region, which is helpful for images with varying lighting conditions (Bhanu & Jing Peng, 2000). The resulting threshold image showed black pixels representing the background (*i.e.*, the sticky trap) and white pixels representing the foreground (*i.e.*, arthropods and everything else that was sticked to the trap). We used a dilation morphological operation in the binarized images to expand the boundaries of the foreground region (Comer, 1999). Next, we extracted the bounding box (*i.e.*, x and y minimum and maximum coordinates) that restricted each foreground region (*i.e.*, each individual) and employed a cluster analysis (using Gower’s distance, see Gower, 1966) to group clusters of white pixels that were close to each other in the image. We then divided the image into smaller pieces corresponding to each group generated by the cluster analysis. All these procedures were carried out using the R software, with the imager (Barthelmé & Tschumperlé, 2019) and EBImage (Pau et al., 2010) packages. After the clustering method, we retained 13431 small pieces of images, from which we selected 1000 random images to use during the training process of our YOLO object detection model.

The model reached a mean box loss of 0.33 (± 0.08), mean classification loss of 0.25 (± 0.09), and a mean Distribution Focal Loss of 0.86 (± 0.06) across the 5 folds after 250 training steps (Figure S2). We calculated the final metrics after validation at the confidence threshold in which the F1 metric was the highest for each group at an Intersection Over Union (IoU) of 0.5 (Table S1). Mean average precision reached 0.74 (± 0.05) for all groups across the five model folds, while recall reached 0.81 (± 0.05) and F1 reached 0.77 (± 0.03; Table S1).


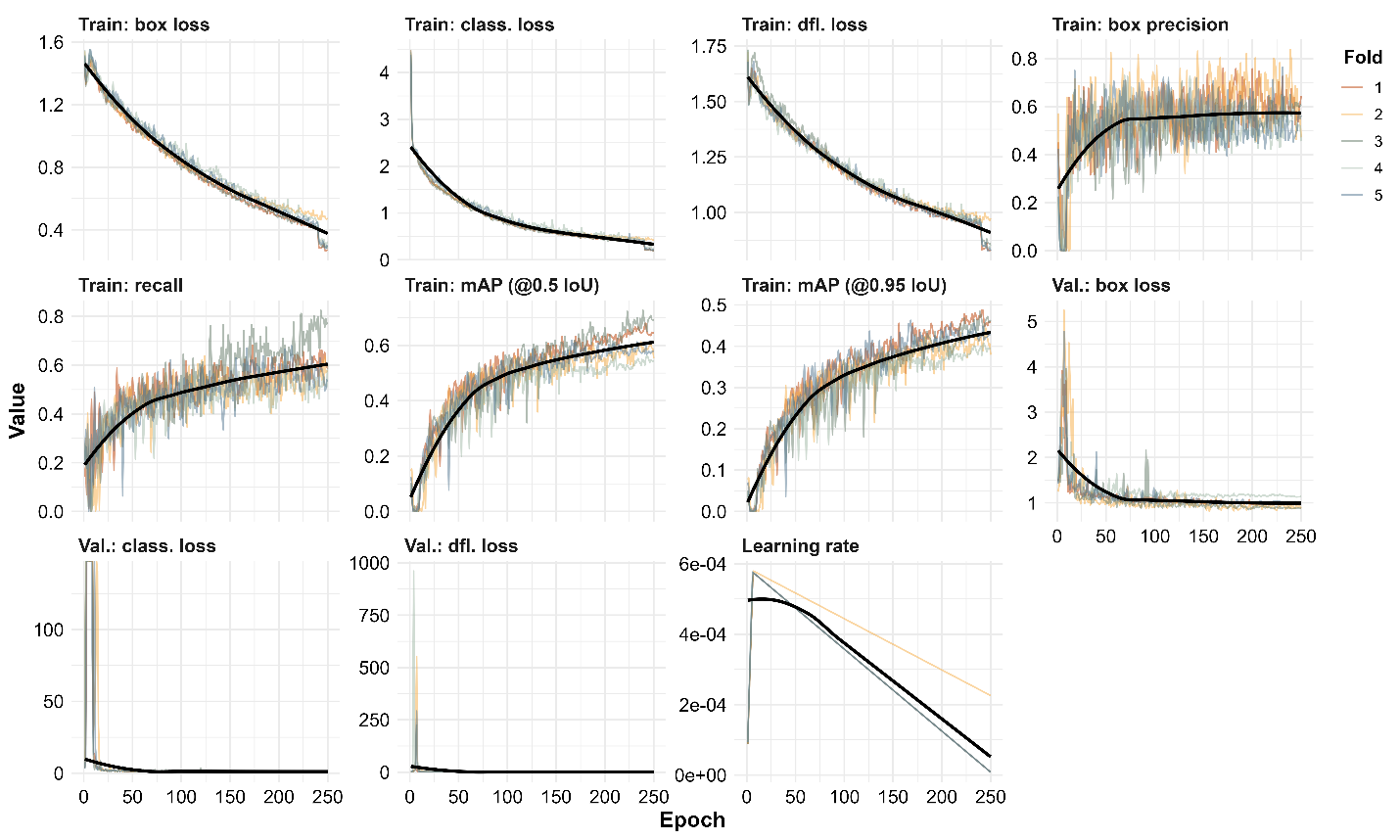


**Figure S2.** Visual representation of the model metrics throughout the 250 steps.

## Table S1. Validation metrics of the model for each class. Average values for all groups per model fold are highlighted in bold.

| **Threshold** | **Precision** | **Recall** | **F1** | **Class** | **Fold** |
| --- | --- | --- | --- | --- | --- |
| **0.6100** | **0.6920** | **0.7692** | **0.7286** | **All groups** | **1** |
| 0.1400 | 0.5294 | 0.7941 | 0.6353 | Brachycera | 1 |
| 0.1700 | 0.6769 | 0.6769 | 0.6769 | Ephemeroptera | 1 |
| 0.5800 | 1.0000 | 1.0000 | 1.0000 | Odonata | 1 |
| 0.0000 | 0.0000 | 0.0000 | 0.0000 | Orthoptera | 1 |
| 0.8200 | 0.9189 | 0.6939 | 0.7907 | Nematocera | 1 |
| 0.6900 | 0.8438 | 0.9310 | 0.8852 | Coleoptera | 1 |
| 0.0400 | 1.0000 | 1.0000 | 1.0000 | Plecoptera | 1 |
| 0.8100 | 1.0000 | 0.5000 | 0.6667 | Araneae | 1 |
| 0.8700 | 0.9118 | 0.8611 | 0.8857 | Hemiptera | 1 |
| 0.9200 | 0.5000 | 1.0000 | 0.6667 | Trichoptera | 1 |
| 0.7100 | 1.0000 | 1.0000 | 1.0000 | Isoptera | 1 |
| 0.1100 | 0.6047 | 0.9630 | 0.7429 | Hymenoptera | 1 |
| 0.3400 | 1.0000 | 0.8333 | 0.9091 | Lepidoptera | 1 |
| **0.3700** | **0.7000** | **0.8634** | **0.7732** | **All groups** | **2** |
| 0.3800 | 0.7297 | 1.0000 | 0.8438 | Brachycera | 2 |
| 0.2400 | 0.6757 | 0.7576 | 0.7143 | Ephemeroptera | 2 |
| 0.3400 | 1.0000 | 1.0000 | 1.0000 | Odonata | 2 |
| 0.0000 | 0.0000 | 0.0000 | 0.0000 | Orthoptera | 2 |
| 0.3700 | 0.8182 | 0.8182 | 0.8182 | Nematocera | 2 |
| 0.7600 | 0.8182 | 0.9000 | 0.8571 | Coleoptera | 2 |
| 0.0000 | 0.5000 | 1.0000 | 0.6667 | Plecoptera | 2 |
| 0.7000 | 0.7500 | 1.0000 | 0.8571 | Araneae | 2 |
| 0.6700 | 0.7619 | 0.8889 | 0.8205 | Hemiptera | 2 |
| 0.0400 | 0.7143 | 1.0000 | 0.8333 | Trichoptera | 2 |
| 0.0000 | 0.0000 | 0.0000 | 0.0000 | Isoptera | 2 |
| 0.7400 | 0.7500 | 0.8571 | 0.8000 | Hymenoptera | 2 |
| 0.8800 | 1.0000 | 0.5000 | 0.6667 | Lepidoptera | 2 |
| **0.3800** | **0.7845** | **0.8706** | **0.8253** | **All groups** | **3** |
| 0.0700 | 0.6923 | 0.9310 | 0.7941 | Brachycera | 3 |
| 0.2200 | 0.8750 | 0.7609 | 0.8140 | Ephemeroptera | 3 |
| 0.0600 | 0.8000 | 1.0000 | 0.8889 | Odonata | 3 |
| 0.0000 | 0.0000 | 0.0000 | 0.0000 | Orthoptera | 3 |
| 0.0600 | 0.7193 | 0.8723 | 0.7885 | Nematocera | 3 |
| 0.4200 | 0.9070 | 1.0000 | 0.9512 | Coleoptera | 3 |
| 0.0900 | 1.0000 | 1.0000 | 1.0000 | Plecoptera | 3 |
| 0.0200 | 0.2857 | 1.0000 | 0.4444 | Araneae | 3 |
| 0.5300 | 0.8750 | 0.9130 | 0.8936 | Hemiptera | 3 |
| 0.0600 | 0.6667 | 0.8000 | 0.7273 | Trichoptera | 3 |
| 0.2400 | 0.6667 | 1.0000 | 0.8000 | Isoptera | 3 |
| 0.7900 | 0.7097 | 0.8462 | 0.7719 | Hymenoptera | 3 |
| 0.1100 | 1.0000 | 1.0000 | 1.0000 | Lepidoptera | 3 |
| **0.5700** | **0.7164** | **0.8041** | **0.7577** | **All groups** | **4** |
| 0.1100 | 0.6190 | 0.8667 | 0.7222 | Brachycera | 4 |
| 0.7500 | 0.8214 | 0.6053 | 0.6970 | Ephemeroptera | 4 |
| 0.8500 | 0.7500 | 0.7500 | 0.7500 | Odonata | 4 |
| 0.0000 | 0.0000 | 0.0000 | 0.0000 | Orthoptera | 4 |
| 0.1900 | 0.8028 | 0.9048 | 0.8507 | Nematocera | 4 |
| 0.7000 | 0.8333 | 0.9375 | 0.8824 | Coleoptera | 4 |
| 0.9200 | 1.0000 | 1.0000 | 1.0000 | Plecoptera | 4 |
| 0.0100 | 0.5000 | 1.0000 | 0.6667 | Araneae | 4 |
| 0.5300 | 0.8163 | 0.9302 | 0.8696 | Hemiptera | 4 |
| 0.0100 | 0.5714 | 1.0000 | 0.7273 | Trichoptera | 4 |
| 0.0100 | 0.3333 | 0.5000 | 0.4000 | Isoptera | 4 |
| 0.7400 | 0.5161 | 0.8000 | 0.6275 | Hymenoptera | 4 |
| 0.5700 | 0.7500 | 0.7500 | 0.7500 | Lepidoptera | 4 |
| **0.6900** | **0.8226** | **0.7356** | **0.7766** | **All groups** | **5** |
| 0.3300 | 0.7143 | 0.9259 | 0.8065 | Brachycera | 5 |
| 0.6100 | 0.8824 | 0.6250 | 0.7317 | Ephemeroptera | 5 |
| 0.8200 | 1.0000 | 0.5000 | 0.6667 | Odonata | 5 |
| 0.0000 | 0.0000 | 0.0000 | 0.0000 | Orthoptera | 5 |
| 0.6900 | 0.9310 | 0.6923 | 0.7941 | Nematocera | 5 |
| 0.0200 | 0.8205 | 1.0000 | 0.9014 | Coleoptera | 5 |
| 0.0200 | 0.5000 | 1.0000 | 0.6667 | Plecoptera | 5 |
| 0.0100 | 0.6667 | 1.0000 | 0.8000 | Araneae | 5 |
| 0.2700 | 0.7800 | 0.9512 | 0.8571 | Hemiptera | 5 |
| 0.0200 | 0.6667 | 1.0000 | 0.8000 | Trichoptera | 5 |
| 0.0000 | 0.0000 | 0.0000 | 0.0000 | Isoptera | 5 |
| 0.6800 | 0.7500 | 0.8750 | 0.8077 | Hymenoptera | 5 |
| 0.0300 | 1.0000 | 1.0000 | 1.0000 | Lepidoptera | 5 |

Image annotation was conducted in R using the XML package (Comprehensive R Archive Network Team et al., 2023) and in the LabelImg interface (Tzutalin, 2015) developed in Python ((Van Rossum & Drake Jr, 1995). We trained the model in the Google Colab interface (Bisong, 2019) and the full pipeline to reproduce our analysis is available at https://doi.org/10.5281/zenodo.15238078. Check a visual representation of the steps described here in Figure S3.

##
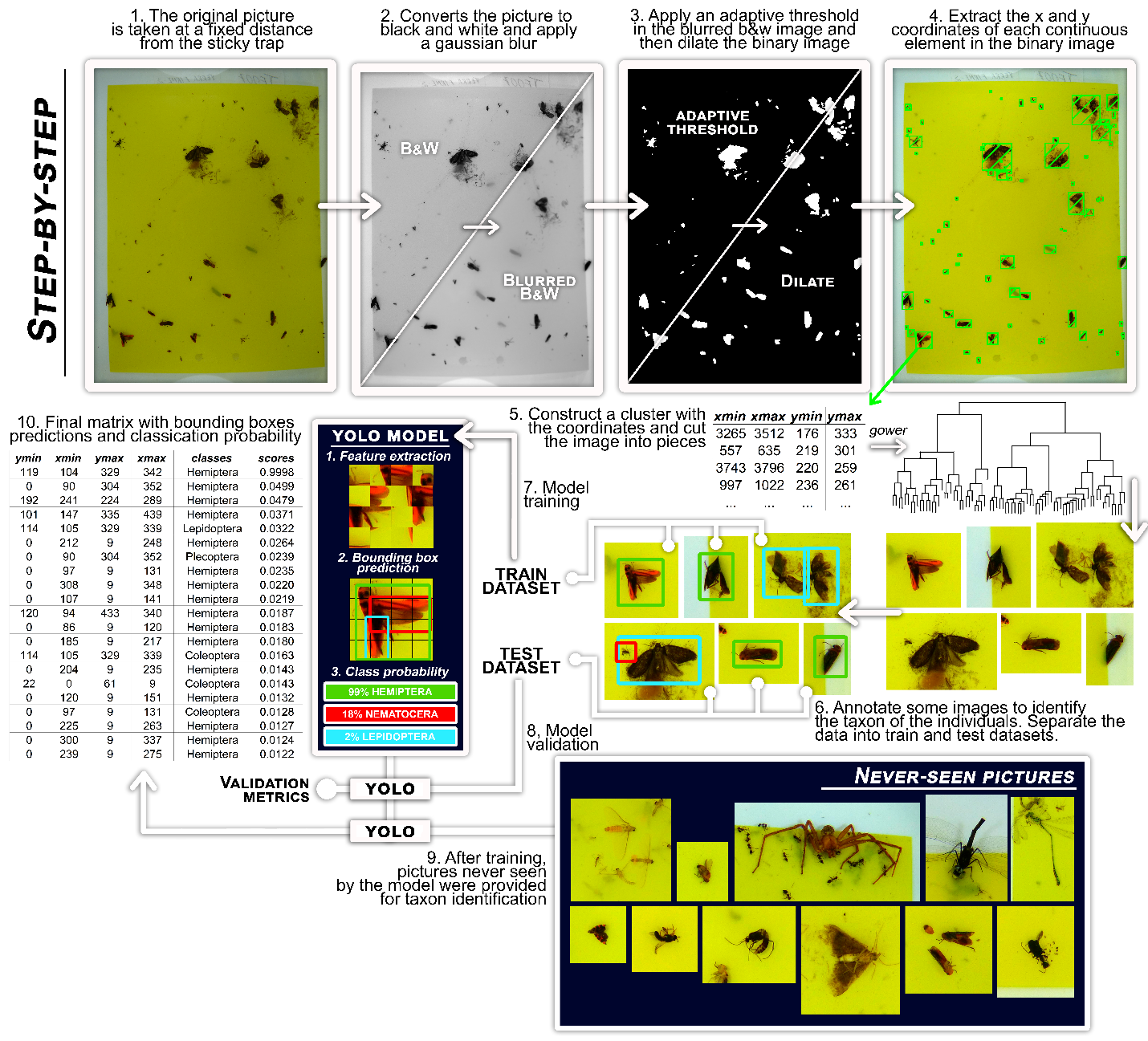
Figure S3. Step by step procedures of image treatment and object detection model training. The original picture is first taken from a fixed distance from the sticky trap. This colour image is then converted to grayscale and a gaussian blur is applied to soften the edges of individuals. Then, we convert the image to binary using an adaptive threshold in the blurred grayscale image. This binary image is then dilated to enlarge the edges of white elements and the x and y coordinates of each continuous white element is retrieved. This coordinates matrix is then used to construct a cluster and group white element close to each other in the same group, generating pieces of images. These pieces are annotated and divided into train, test and remaining images. The train dataset is used to train the YOLO object detection model, whereas the test dataset is used to validate the model and extract the validation metrics. The remaining pictures (i.e., never seen before by the model) are then provided to the model after training and validation, which returns a matrix of the predicted bounding boxes and the probability of the class for each bounding box.

## References

Barthelmé, S., & Tschumperlé, D. (2019). imager: an R package for image processing based on CImg. *Journal of Open Source Software*, *4*(38), 1012. https://doi.org/10.21105/joss.01012

Bhanu, B., & Jing Peng. (2000). Adaptive integrated image segmentation and object recognition. *IEEE Transactions on Systems, Man and Cybernetics, Part C (Applications and Reviews)*, *30*(4), 427–441. https://doi.org/10.1109/5326.897070

Bisong, E. (2019). Google Colaboratory. In *Building Machine Learning and Deep Learning Models on Google Cloud Platform* (pp. 59–64). Apress. https://doi.org/10.1007/978-1-4842-4470-8_7

Comer, M. L. (1999). Morphological operations for color image processing. *Journal of Electronic Imaging*, *8*(3), 279. https://doi.org/10.1117/1.482677

Comprehensive R Archive Network Team, Duncan Temple Lang, & Tomas Kalibera. (2023). *Tools for Parsing and Generating XML Within R and S-Plus*. https://www.omegahat.net/RSXML/

Gower, J. C. (1966). Some distance properties of latent root and vector methods used in multivariate analysis. *Biometrika*, *53*(3–4), 325–338. https://doi.org/10.1093/biomet/53.3-4.325

Pau, G., Fuchs, F., Sklyar, O., Boutros, M., & Huber, W. (2010). EBImage—an R package for image processing with applications to cellular phenotypes. *Bioinformatics*, *26*(7), 979–981. https://doi.org/10.1093/bioinformatics/btq046

Tzutalin, D. (2015). LabelImg. In *GitHub repository* (Vol. 6). https://github.com/heartexlabs/labelImg

Van Rossum, G., & Drake Jr, F. L. (1995). *Python reference manual*. Centrum voor Wiskunde en Informatica Amsterdam.

**Appendix S3.** Examples of detections done by our trained model. Color of the boxes represent different taxa, which is identified above each bounding box with the respective class probability value.


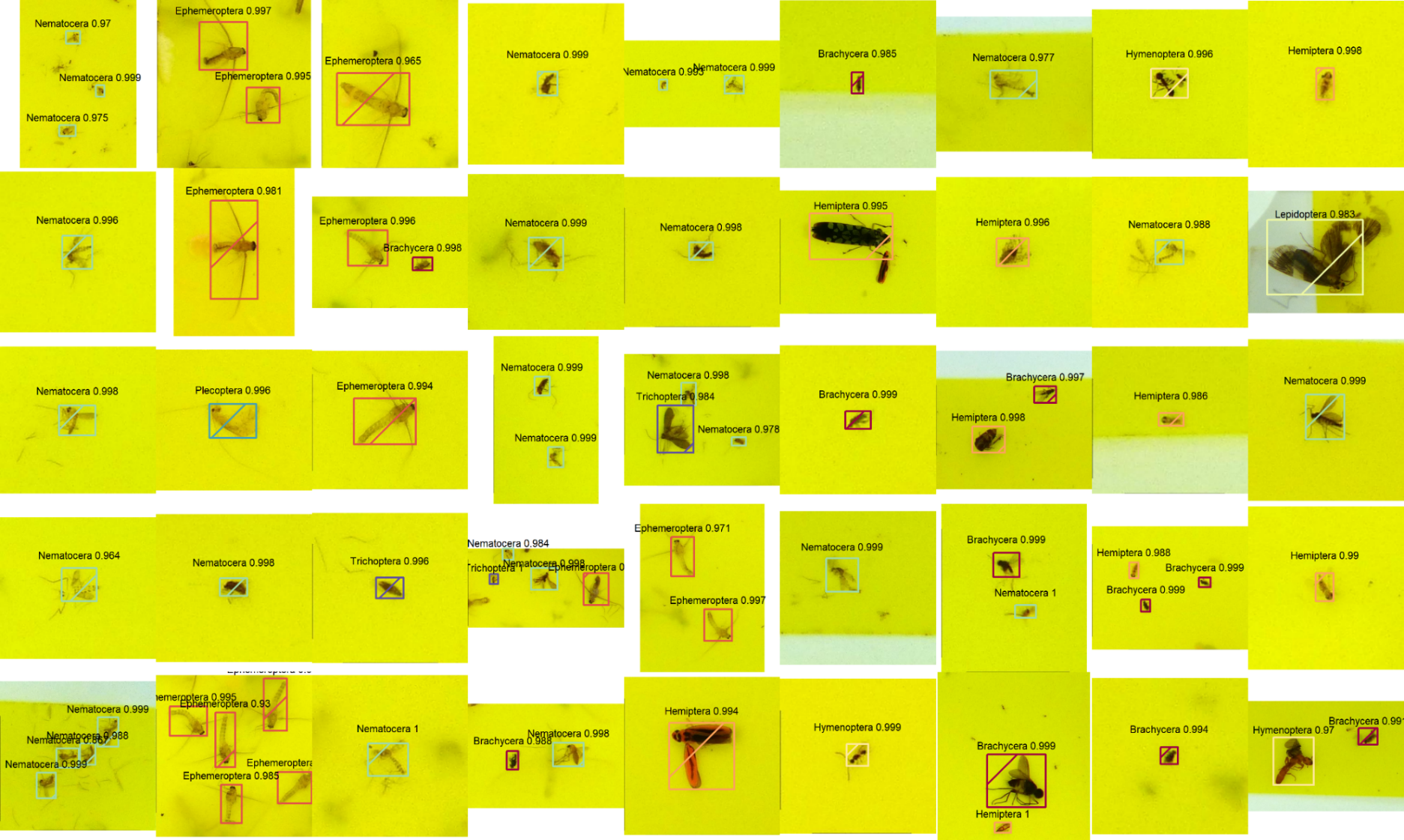


**Figure S4.** Examples of detections done by our trained model. Color of the boxes represent different taxa, which is identified above each bounding box with the respective class probability value

**Appendix S4.** Moran’s I result.

**Table S2.** Moran’s I coefficients for both lifecycle (i.e., terrestrial and aquatic) and the specific taxonomical groups. We present the expected and observed Moran’s I values, its respective standard deviation (SD), P values, and spatial scales for richness, composition, abundance and body size.

| **Group** | **Observed** | **Expected** | **SD** | **P** | **Response** |
| --- | --- | --- | --- | --- | --- |
| Brachycera | -0.000162 | -0.004292 | 0.002788 | 0.138491 | Abundance |
| Coleoptera | -0.003615 | -0.004292 | 0.002697 | 0.801762 | Abundance |
| Ephemeroptera | -0.003253 | -0.004292 | 0.001424 | 0.465601 | Abundance |
| Hemiptera | -0.002395 | -0.004292 | 0.002301 | 0.409677 | Abundance |
| Hymenoptera | -0.000516 | -0.004292 | 0.002823 | 0.181085 | Abundance |
| Nematocera | -0.002141 | -0.004292 | 0.002833 | 0.447627 | Abundance |
| Trichoptera | -0.000725 | -0.004292 | 0.002727 | 0.190873 | Abundance |
| Brachycera | -0.001349 | -0.004785 | 0.00311 | 0.269155 | Body size |
| Coleoptera | -0.002849 | -0.00625 | 0.003748 | 0.364157 | Body size |
| Ephemeroptera | -0.006092 | -0.019608 | 0.012287 | 0.271309 | Body size |
| Hemiptera | -0.003153 | -0.006579 | 0.004123 | 0.406102 | Body size |
| Hymenoptera | -0.044992 | -0.007194 | 0.00416 | 0 | Body size |
| Nematocera | -0.002522 | -0.004405 | 0.002844 | 0.507802 | Body size |
| Trichoptera | -0.002073 | -0.007463 | 0.005004 | 0.281459 | Body size |
| Lifecycle | -0.00088 | -0.005376 | 0.003527 | 0.202359 | Abundance-based composition |
| Lifecycle | -0.00188 | -0.005376 | 0.003546 | 0.324083 | Occurrence-based composition |
| Lifecycle | -0.000476 | -0.002674 | 0.001791 | 0.219736 | N-component |
| Lifecycle | -0.000545 | -0.002674 | 0.001771 | 0.229373 | SAD-component |

**Appendix S5.** Generalized Additive Models coefficients for the two diversity components (i.e., SAD and N) and composition of different lifecycle groups at nine landscape sizes.

**Table S3.** Coefficients of the Generalized Additive Models for the fixed effects of forest cover and random effects of spatial coordinates on SAD-component, N-component, abundance-based composition, and occurrence-based composition of different lifecycle groups. We presented estimate, statistics (i.e., *z*, *t* or *x²*, depending on data distribution), adjusted r-squared (R2), Akaike Information Criteria (AIC), k-not value and P values for fixed and random effects. Values of significance (Signif.) refer to P values and are as follows: “****” is P < 1e-04, “***” is P < 0.001, “**” is P < 0.01, “*” P < 0.05, “.” P < 0.1, and “ns” is P > 0.1.

| Response | Effect type | Explanatory | Estimate | Statistics | K | P | Signif | R2 | AIC |
| --- | --- | --- | --- | --- | --- | --- | --- | --- | --- |
| Abundance-based composition | Fixed effects | (Intercept) | -0.382 | -15.277 | 7 | 3.31E-34 | **** | 0.77 | -141.362 |
| Abundance-based composition | Fixed effects | Area | 0.74 | 21.911 | 7 | 2.05E-52 | **** | 0.77 | -141.362 |
| Abundance-based composition | Random effects | Coordinates | NA | 20.648 | 7 | 0.008570812 | ** | 0.77 | -141.362 |
| N-component | Fixed effects | (Intercept) | 1.099 | 17.831 | 7 | 1.27E-51 | **** | 0.253 | 639.298 |
| N-component | Fixed effects | Area | -0.928 | -9.057 | 7 | 8.17E-18 | **** | 0.253 | 639.298 |
| N-component | Fixed effects | Life cycle | -0.853 | -8.736 | 7 | 8.82E-17 | **** | 0.253 | 639.298 |
| N-component | Fixed effects | Area:Life cycle | 1.356 | 9.724 | 13 | 4.99E-20 | **** | 0.253 | 639.298 |
| N-component | Random effects | Coordinates | NA | 29.407 | 13 | 7.82E-05 | **** | 0.253 | 639.298 |
| Occurrence-based composition | Fixed effects | (Intercept) | -0.116 | -6.244 | 12 | 2.90E-09 | **** | 0.427 | -219.006 |
| Occurrence-based composition | Fixed effects | Area | 0.287 | 11.848 | 12 | 2.11E-24 | **** | 0.427 | -219.006 |
| Occurrence-based composition | Random effects | Coordinates | NA | 0.801 | 12 | 0.670511487 | ns | 0.427 | -219.006 |
| SAD-component | Fixed effects | (Intercept) | 1.064 | 11.432 | 14 | 4.39E-26 | **** | 0.495 | 949.14 |
| SAD-component | Fixed effects | Area | 0.464 | 3 | 14 | 0.002883203 | ** | 0.495 | 949.14 |
| SAD-component | Fixed effects | Life cycle | 1.604 | 10.862 | 14 | 5.24E-24 | **** | 0.495 | 949.14 |
| SAD-component | Fixed effects | Area:Life cycle | -0.089 | -0.424 | 13 | 0.672047859 | ns | 0.495 | 949.14 |
| SAD-component | Random effects | Coordinates | NA | 18.141 | 13 | 0.006189961 | ** | 0.495 | 949.14 |

**Appendix S6.** Generalized Additive Mixed Models coefficients for abundance and body size of different taxonomic groups at nine landscape sizes.

**Table S4.** Coefficients of the Generalized Additive Mixed Models for the fixed effects of forest cover and random effects of spatial coordinates on the abundance and body size of the specific taxonomic groups (i.e., bees and wasps, beetles, caddisflies, cicadas, flies, and mosquitoes). We presented estimate, statistics (i.e., *z*, *t* or *x²*, depending on data distribution), number of knots (K), adjusted r-squared (R2), Akaike Information Criteria (AIC) and P values for fixed and random effects at nine different spatial scales. Appropriate scales of effect for each response variable are presented in bold. Values of significance (Signif.) refer to P values and are as follows: “****” is P < 1e-04, “***” is P < 0.001, “**” is P < 0.01, “*” P < 0.05, “.” P < 0.1, and “ns” is P > 0.1.

| **Response** | **Taxon** | **Effect type** | **Explanatory** | **Estimate** | **Statistics** | **K** | **P** | **Signif** | **R2** | **AIC** |
| --- | --- | --- | --- | --- | --- | --- | --- | --- | --- | --- |
| Abundance | Brachycera | Fixed effects | (Intercept) | 1.1598 | 18.5434 | 20 | 9.23E-77 | **** | 0.0096 | 1773.834072 |
| Abundance | Brachycera | Fixed effects | Area | 0.6528 | 7.4748 | 20 | 7.73E-14 | **** | 0.0096 | 1773.834072 |
| Abundance | Brachycera | Random effects | Coordinates | NA | 153.9715 | 20 | 0 | **** | 0.0096 | 1773.834072 |
| Abundance | Coleoptera | Fixed effects | (Intercept) | 0.3672 | 4.2224 | 19 | 2.42E-05 | **** | 0.092 | 1558.750577 |
| Abundance | Coleoptera | Fixed effects | Area | 1.2016 | 11.121 | 19 | 9.92E-29 | **** | 0.092 | 1558.750577 |
| Abundance | Coleoptera | Random effects | Coordinates | NA | 181.9918 | 19 | 0 | **** | 0.092 | 1558.750577 |
| Abundance | Ephemeroptera | Fixed effects | (Intercept) | -2.1113 | -6.3223 | 19 | 2.58E-10 | **** | 0.0232 | 10734.28334 |
| Abundance | Ephemeroptera | Fixed effects | Area | -0.4078 | -6.9916 | 19 | 2.72E-12 | **** | 0.0232 | 10734.28334 |
| Abundance | Ephemeroptera | Random effects | Coordinates | NA | 2518.9165 | 19 | 0 | **** | 0.0232 | 10734.28334 |
| Abundance | Hemiptera | Fixed effects | (Intercept) | -0.2417 | -2.0675 | 19 | 0.038686011 | * | 0.7323 | 1296.917851 |
| Abundance | Hemiptera | Fixed effects | Area | 2.3703 | 17.4031 | 19 | 7.82E-68 | **** | 0.7323 | 1296.917851 |
| Abundance | Hemiptera | Random effects | Coordinates | NA | 655.2443 | 19 | 0 | **** | 0.7323 | 1296.917851 |
| Abundance | Hymenoptera | Fixed effects | (Intercept) | -0.2199 | -1.8888 | 18 | 0.058921873 | . | 0.1446 | 937.7222775 |
| Abundance | Hymenoptera | Fixed effects | Area | 1.1529 | 7.6808 | 18 | 1.58E-14 | **** | 0.1446 | 937.7222775 |
| Abundance | Hymenoptera | Random effects | Coordinates | NA | 61.5005 | 18 | 2.79E-06 | **** | 0.1446 | 937.7222775 |
| Abundance | Nematocera | Fixed effects | (Intercept) | 4.9989 | 255.5034 | 20 | 0 | **** | 0.6896 | 5851.71483 |
| Abundance | Nematocera | Fixed effects | Area | -3.1145 | -78.647 | 20 | 0 | **** | 0.6896 | 5851.71483 |
| Abundance | Nematocera | Random effects | Coordinates | NA | 3353.3889 | 20 | 0 | **** | 0.6896 | 5851.71483 |
| Abundance | Trichoptera | Fixed effects | (Intercept) | 0.7257 | 7.22 | 19 | 5.20E-13 | **** | 0.2509 | 774.1385623 |
| Abundance | Trichoptera | Fixed effects | Area | -1.2018 | -6.8714 | 19 | 6.36E-12 | **** | 0.2509 | 774.1385623 |
| Abundance | Trichoptera | Random effects | Coordinates | NA | 92.0399 | 19 | 0 | **** | 0.2509 | 774.1385623 |
| Body size | Brachycera | Fixed effects | (Intercept) | 11.1121 | 87.5853 | 1 | 1.45E-164 | **** | 0.0167 | NA |
| Body size | Brachycera | Fixed effects | Area | -0.3488 | -1.7424 | 1 | 0.082943032 | . | 0.0167 | NA |
| Body size | Brachycera | Random effects | Coordinates | NA | 2.9223 | 1 | 0.354888747 | ns | 0.0167 | NA |
| Body size | Coleoptera | Fixed effects | (Intercept) | 12.3488 | 57.2055 | 1 | 6.90E-107 | **** | -0.0015 | NA |
| Body size | Coleoptera | Fixed effects | Area | -0.6163 | -1.7242 | 1 | 0.086643078 | . | -0.0015 | NA |
| Body size | Coleoptera | Random effects | Coordinates | NA | 0.4332 | 1 | 0.922062892 | ns | -0.0015 | NA |
| Body size | Ephemeroptera | Fixed effects | (Intercept) | 13.9179 | 42.9026 | 1 | 1.95E-39 | **** | 0.0863 | NA |
| Body size | Ephemeroptera | Fixed effects | Area | -1.0977 | -2.0084 | 1 | 0.050342672 | . | 0.0863 | NA |
| Body size | Ephemeroptera | Random effects | Coordinates | NA | 4.8736 | 1 | 0.184391011 | ns | 0.0863 | NA |
| Body size | Hemiptera | Fixed effects | (Intercept) | 12.4876 | 108.9403 | 1 | 1.50E-143 | **** | 0.0618 | NA |
| Body size | Hemiptera | Fixed effects | Area | -0.1195 | -0.6734 | 1 | 0.501759928 | ns | 0.0618 | NA |
| Body size | Hemiptera | Random effects | Coordinates | NA | 13.5497 | 1 | 0.004804592 | ** | 0.0618 | NA |
| Body size | Hymenoptera | Fixed effects | (Intercept) | 12.4388 | 49.0839 | 1 | 5.63E-88 | **** | 0.0853 | NA |
| Body size | Hymenoptera | Fixed effects | Area | -0.563 | -1.7343 | 1 | 0.085141616 | . | 0.0853 | NA |
| Body size | Hymenoptera | Random effects | Coordinates | NA | 21.0497 | 1 | 0.00020894 | *** | 0.0853 | NA |
| Body size | Nematocera | Fixed effects | (Intercept) | 11.6451 | 113.2068 | 1 | 3.68E-199 | **** | 0.0181 | NA |
| Body size | Nematocera | Fixed effects | Area | 0.0598 | 0.4087 | 1 | 0.68317938 | ns | 0.0181 | NA |
| Body size | Nematocera | Random effects | Coordinates | NA | 6.0203 | 1 | 0.087812015 | . | 0.0181 | NA |
| Body size | Trichoptera | Fixed effects | (Intercept) | 8.9601 | 64.2771 | 1 | 6.13E-101 | **** | 0.0346 | NA |
| Body size | Trichoptera | Fixed effects | Area | -0.1405 | -0.6899 | 1 | 0.491482189 | ns | 0.0346 | NA |
| Body size | Trichoptera | Random effects | Coordinates | NA | 7.7061 | 1 | 0.02366348 | * | 0.0346 | NA |
